# Association of shorter leucocyte telomere length with risk of frailty

**DOI:** 10.1002/jcsm.12971

**Published:** 2022-03-17

**Authors:** Vasiliki Bountziouka, Christopher P. Nelson, Veryan Codd, Qingning Wang, Crispin Musicha, Elias Allara, Stephen Kaptoge, Emanuele Di Angelantonio, Adam S. Butterworth, John R. Thompson, Elizabeth M. Curtis, Angela M. Wood, John N. Danesh, Nicholas C. Harvey, Cyrus Cooper, Nilesh J. Samani

**Affiliations:** ^1^ Department of Cardiovascular Sciences University of Leicester Leicester UK; ^2^ NIHR Leicester Biomedical Research Centre, Glenfield Hospital Leicester UK; ^3^ British Heart Foundation Cardiovascular Epidemiology Unit, Department of Public Health and Primary Care University of Cambridge Cambridge UK; ^4^ National Institute for Health Research Blood and Transplant Research Unit in Donor Health and Genomics, University of Cambridge Cambridge UK; ^5^ British Heart Foundation Centre of Research Excellence University of Cambridge Cambridge UK; ^6^ Health Data Research UK Cambridge, Wellcome Genome Campus and University of Cambridge Cambridge UK; ^7^ Department of Health Sciences University of Leicester Leicester UK; ^8^ MRC Lifecourse Epidemiology Unit University of Southampton Southampton UK; ^9^ Wellcome Sanger Institute, Wellcome Genome Campus Cambridge Hinxton UK; ^10^ NIHR Southampton Biomedical Research Centre University of Southampton and University Hospital Southampton NHS Foundation Trust Southampton UK; ^11^ NIHR Biomedical Research Centre University of Oxford Oxford UK

**Keywords:** Leucocyte telomere length, Frailty, Biological age, UK biobank

## Abstract

**Background:**

Frailty is a multidimensional syndrome of decline that affects multiple systems and predisposes to adverse health outcomes. Although chronological age is the major risk factor, inter‐individual variation in risk is not fully understood. Leucocyte telomere length (LTL), a proposed marker of biological age, has been associated with risk of many diseases. We sought to determine whether LTL is associated with risk of frailty.

**Methods:**

We utilized cross‐sectional data from 441 781 UK Biobank participants (aged 40–69 years), with complete data on frailty indicators and LTL. Frailty was defined as the presence of at least three of five indicators: weaker grip strength, slower walking pace, weight loss in the past year, lower physical activity, and exhaustion in the past 2 weeks. LTL was measured using a validated qPCR method and reported as a ratio of the telomere repeat number (T) to a single‐copy gene (S) (T/S ratio). Association of LTL with frailty was evaluated using adjusted (chronological age, sex, deprivation, smoking, alcohol intake, body mass index, and multimorbidity) multinomial and ordinal regression models, and results are presented as relative risk (RRR) or odds ratios (OR), respectively, alongside the 95% confidence interval (CI). Mendelian randomization (MR), using 131 genetic variants associated with LTL, was used to assess if the association of LTL with frailty was causal.

**Results:**

Frail participants (4.6%) were older (median age difference (95% CI): 3 (2.5; 3.5) years, *P* = 2.73 × 10^−33^), more likely to be female (61%, *P* = 1.97 × 10^−129^), and had shorter LTL (−0.13SD vs. 0.03SD, *P* = 5.43 × 10^−111^) than non‐frail. In adjusted analyses, both age and LTL were associated with frailty (RRR = 1.03 (95% CI: 1.02; 1.04) per year of older chronological age, *P* = 3.99 × 10^−12^; 1.10 (1.08; 1.11) per SD shorter LTL, *P* = 1.46 × 10^−30^). Within each age group (40–49, 50–59, 60–69 years), the prevalence of frailty was about 33% higher in participants with shorter (−2SD) versus longer telomeres (+2SD). MR analysis showed an association of LTL with frailty that was directionally consistent with the observational association, but not statistically significant (MR‐Median: OR (95% CI): 1.08 (0.98; 1.19) per SD shorter LTL, *P* = 0.13).

**Conclusions:**

Inter‐individual variation in LTL is associated with the risk of frailty independently of chronological age and other risk factors. Our findings provide evidence for an additional biological determinant of frailty.

## Introduction

Frailty is a multidimensional syndrome of decline that affects multiple systems and predisposes to adverse health outcomes.^1^ It is associated with greater vulnerability to stressors and increased risk of adverse health outcomes, including falls, fractures, hospitalization, and death.^2^ Frailty is inter‐related, but not synonymous, with co‐morbidity and disability. Approaches to its operational definition include an accumulation of deficits as proposed by Rockwood *et al*.,^1^ or a specific biological syndrome, characterized by weight loss, fatigue, reduced muscle strength, reduced walking speed, and low physical activity, as proposed by Fried.^3^ Both definitions have strengths and weaknesses: the Fried physical frailty phenotype includes two components of sarcopenia and may therefore overlap considerably with muscle function; the Rockwood approach essentially assesses the number of co‐morbidities, with its attendant circularity of cause and effect. Attempts have been made to achieve consensus on the definition of frailty, recognizing that it is characterized by a plethora of physical, psychological, physiological, and social life aspects that co‐exist in complex combinations.^4^, ^5^, ^6^, ^7^

Although frailty is more prevalent in older people, it does not occur exclusively above any specific chronological age threshold.^8^ Hence, there is a need to identify other biological factors that may predispose to frailty. There is a particular interest in whether biological age, as distinct from chronological age, is associated with risk of frailty.^9^ Telomere length has emerged as a potential biomarker of biological age, with shorter telomeres indicating more advanced biological age.^10^ Shorter mean leucocyte telomere length (LTL) has been associated with risk of several age‐associated diseases with causal inference analyses suggesting that some of the associations are primary.^11^, ^12^ However, it should be noted that the relationship between LTL and disease is complex and longer LTL can also be associated with disease risk, most notably for several cancers.^11^, ^12^, ^13^ Current evidence on whether inter‐individual variation in LTL is associated with higher risk of frailty is inconclusive.^14^, ^15^, ^16^

We have recently generated cohort‐wide LTL measurements in UK Biobank (UKB).^17^ Using this large‐scale resource, we investigated whether LTL is associated with frailty independently of chronological age and other established risk factors.

## Materials and methods

### Participants and data collection

As previously described,^18^ UKB recruited 502 478 participants aged 40–69 years during the years 2006–2010. Participants have been characterized in detail using questionnaires, physical measurements, biological assays, and longitudinal linkage with multiple health record systems. Detailed information regarding the physical assessments undertaken is available at https://www.ukbiobank.ac.uk/. UKB received approval from the North West Centre for Research Ethics Committee (11/NW/0382) and have therefore been performed in accordance with the ethical standards laid down in the 1964 Declaration of Helsinki and its later amendments. The use of data presented in this paper was approved by the Access Committee of UKB under application number 6077.

### Frailty phenotype

Based on the concept of frailty as a biological syndrome, we implemented the ‘phenotype’ model of frailty, developed by Fried *et al*.,^3^ which has been previously utilized in the UKB.^19^, ^20^ Under this model, five indicators, assessed at baseline examination, were used to define frailty: weakness, slowness, weight loss, low physical activity, and exhaustion. For each indicator, we employed the following scoring system: (i) weakness, measured using the maximum hand grip strength from both arms (UKB field codes: ‘46’ and ‘47’): participants in the lowest 20% of the cohort (sex and body mass index (BMI) adjusted) were considered to meet the frailty criteria and thus given a score of ‘1’ or ‘0’ otherwise; (ii) slowness, measured using the self‐reported walking pace (field code: ‘924’): ‘1’ for slow pace, ‘0’ otherwise; (iii) weight loss, measured through the self‐reported weight change (field code: ‘2306’) compared with 1 year ago: ‘1’ for yes‐weight loss, ‘0’ otherwise; (iv) low physical activity, measured through self‐reported types of physical activity (field code: ‘6164’) in the last 4 weeks: ‘1’ for non or light activity (e.g. pruning and watering the lawn), ‘0’ otherwise (e.g. weeding, lawn mowing, carpentry and digging, walking for pleasure, swimming, cycling, or other strenuous sports), and (v) exhaustion, measured through self‐reported tiredness/lethargy in last 2 weeks (field code: ‘2080’): ‘1’ for more than half the days or nearly every day, ‘0’ otherwise.

Participants who responded ‘Do not know’ or ‘Prefer not to answer’ to any of the five frailty indicators, or with missing values, were excluded from the analysis. People with one or two indicators in aggregate were classified as pre‐frail, while frailty was defined as the presence of three or more of the five indicators.

### Leucocyte telomere length measurement

Leucocyte telomere length measurements were undertaken on DNA collected at baseline and quality controlled as described elsewhere.^17^ Briefly, LTL was measured using a validated qPCR method and reported as a ratio of the telomere repeat number (T) to a single‐copy gene (S) (T/S ratio). The measurements were log_e_‐transformed to approximate the normal distribution. We utilized *z*‐standardized values of LTL (UKB field code: ‘22192’) to facilitate comparison with other datasets.^17^

### Other phenotypes

To adjust for other known or potential determinants of frailty,^3^ we extracted information on the following phenotypes also collected at baseline: social deprivation score (based on fifths of Townsend Index deprivation score at the time of recruitment (field code: ‘189’), derived from the 2011 Census UK data,^21^ with 1st fifth being the least deprived), smoking (self‐reported field code ‘20116’ and classified as non‐smoker; ex‐smoker; current smoker), alcohol intake (self‐reported frequency of alcohol intake (field code ‘1558’) and classified as never/special occasions only; 1–3 times per month; 1–4 times per week; daily/almost daily), body mass index [field code ‘21001’ categorized as underweight (<18.5 kg/m^2^); normal weight (18.5–24.9 kg/m^2^); overweight (25–29.9 kg/m^2^); obese (≥30 kg/m^2^)] and multimorbidity,^22^ measured as the total number of additional long‐term medical conditions [LTC; pooled out from the self‐reported non‐cancer illness code (field code ‘20002’) and the cancer diagnosed by doctor code (field code ‘2453’)] and classified as none; one LTC; two LTC; three LTC; four or more LTC.

### Statistical analysis

Descriptive statistics are shown as mean (SD), median (1st quartile, 3rd quartile), or frequencies (%). For primary analysis, we used multinomial logistic regression models to investigate the association of chronological age and LTL with frailty status, defined as non‐frail, pre‐frail, and frail. Interaction and quadratic terms for age, LTL, and sex were tested, and the model with the lowest Bayesian information criterion (BIC) was selected. The best model was then adjusted for other potential determinants of frailty. Results are shown as relative risk ratios (RRR) along with their corresponding 95% confidence intervals (95% CI). The average adjusted prediction for the frequency of frailty was plotted against chronological age and LTL. We also report observed associations of the average (‘usual’) LTL values, adjusted for the regression dilution ratio (RDR) of 0.68 (0.64; 0.72) for log_e_‐LTL that was derived using 1351 serial measurements of LTL taken at mean interval of 5.5 years (range: 2–10 years).^17^ Secondary analysis involved multinomial regression and binary logistic regression to assess associations [RRR and odds ratios (OR)] with the number of frailty indicators and the individual frailty indicators, respectively.

We have previously characterized the association of LTL with 123 diseases, identified using hospital admissions, operations, death registry, and self‐report data as described elsewhere.^12^ To assess the extent to which any association of LTL with frailty is independent of any association of LTL with these diseases at baseline, we conducted regression modelling using standardized residuals after regressing LTL on indicators of history of the 123 diseases.

To investigate whether any relationship between LTL and frailty is causal, we conducted Mendelian randomization (MR) analyses,^23^ using 131 independent and uncorrelated genetic variants associated with LTL at genome‐wide significance^12^ as instrumental variables. Further details for the statistical analysis, including the MR analysis, are provided in the ‘Methods section’ of the supporting information.

## Results

Of the 472 174 participants in UK Biobank with a valid LTL measurement, we excluded 30 393 (6.4%) from the current analysis because they lacked information on frailty indicators, relevant covariates, or both (*Figure* S1). There was no difference in the distribution of sex and age between participants who were included or excluded from the analysis (females 54.2% vs. 54.6%; mean age 56.5 vs. 57, respectively). However, those who were excluded had on average shorter telomeres compared to the complete cases for the analysis (−0.019SD vs. 0.001SD).

Of the 441 781 participants included in the analysis, 223 648 (51%) had no frailty indicators, 147 789 (33%) had one indicator, 49 826 (11%) had two indicators, 15 387 (3.5%) had three indicators, 4473 (1%) had four indicators, and 658 (0.15%) had all five indicators. Hence, 20 518 (4.6%) participants met the criteria for frailty and 197 615 (44.7%) for pre‐frailty (*Table* 1). Compared with non‐frail participants, frail participants were older and more likely to be female, socioeconomically deprived, current smokers, obese, alcohol drinkers and report multiple LTCs (*Table* 1). Compared with non‐frail participants, mean LTL was shorter in both frail and in pre‐frail participants, with a greater magnitude of difference for frail participants (*Table* 1).

**Table 1:** Distribution of demographic and clinical characteristics and leucocyte telomere length (LTL), overall and across participants' frailty status

	Frailty status		
	Non‐frail	Pre‐frail	Frail
		(1–2 indicators)	(3–5 indicators)
*n* (%)	223 648 (51)	197 615 (45)	20 518 (4.6)
Age, years	57 (49; 62)	59 (51; 64)	60 (53; 64)
Women, *n* (%)	115 835 (52)	111 119 (56)	12 436 (61)
LTL, SD	0.03 (0.99)	−0.02 (1.00)	−0.13 (1.00)
Fifths of deprivation, *n* (%)			
1st (least deprived)	87 138 (39)	63 773 (32)	4084 (20)
2nd	50 480 (23)	41 727 (21)	3383 (16)
3rd	36 698 (16)	33 617 (17)	3327 (16)
4th	30 047 (13)	31 990 (16)	4389 (21)
5th (most deprived)	19 285 (8.6)	26 508 (13)	5335 (26)
Smoking status, *n* (%)			
Never	126 833 (57)	105 566 (53)	9264 (45)
Previous	77 617 (35)	69 432 (35)	7172 (35)
Current	19 198 (8.6)	22 617 (11)	4082 (20)
Frequency of alcohol intake, *n* (%)			
Daily	51 759 (23)	37 374 (19)	2484 (12)
1–4 times/week	118 555 (53)	92 804 (47)	6745 (33)
1–3 times/month	23 015 (10)	23 627 (12)	2461 (12)
Occasionally/never	30 319 (14)	43 810 (22)	8828 (43)
Categories of body mass index, *n* (%)			
<18 kg/m^2^	1071 (0.48)	992 (0.50)	160 (0.78)
18–25 kg/m^2^	81 510 (36)	58 962 (30)	4138 (20)
25–30 kg/m^2^	98 727 (44)	82 959 (42)	6707 (33)
≥30 kg/m^2^	42 340 (19)	54 702 (28)	9513 (46)
Number of co‐morbidities, *n* (%)			
None	66 333 (30)	39 732 (20)	1307 (6.4)
One long‐term condition	65 376 (29)	50 320 (25)	2690 (13)
Two long‐term conditions	44 089 (20)	41 618 (21)	3483 (17)
Three long‐term conditions	24 672 (11)	28 595 (14)	3563 (17)
Four or more long‐term conditions	23 178 (10)	37 350 (19)	9475 (46)

Results shown as mean (SD) or median (1^st^ quartile; 3^rd^ quartile), unless otherwise indicated. Leucocyte telomere length (LTL) measurements are *z*‐standardized.

Shorter LTL was associated with higher odds of having each of the individual frailty indicators (*Table* 2). Similarly, when participants were dichotomized by the number of frailty indicators, we observed that shorter LTL was associated with higher relative risk of having greater number of frailty indicators (*Table* S1). In analyses subdivided by age and sex (*Table* 3), we found higher prevalence of pre‐frailty and frailty at older ages for both men and women. Mean LTL was higher in women than men, lower at older ages for both sexes, and declined with frailty across all age groups (*Table* 3).

**Table 2:** Adjusted odds ratios (OR) and 95% confidence intervals (95% CI) from a binary logistic regression model of leucocyte telomere length on frailty indicators

	*n* (%)	OR	(95% CI)	*P*
*Weakness (hand grip strength)*	101 179 (23)			
Telomere length, per SD shorter		1.02	(1.01; 1.03)	<0.0001
*Slowness (walking pace)*	34 421 (7.8)			
Telomere length, per SD shorter		1.06	(1.05; 1.07)	<0.0001
*Weight loss*	67 684 (15)			
Telomere length, per SD shorter		1.02	(1.01; 1.03)	<0.0001
*Low physical activity*	56 805 (13)			
Telomere length, per SD shorter		1.04	(1.03; 1.05)	<0.0001
*Exhaustion (tiredness/lethargy)*	54 695 (12)			
Telomere length, per SD shorter		1.03	(1.02; 1.04)	<0.0001

Models additionally adjusted for age, sex, fifths of Townsend index of deprivation (2011), smoking, alcohol intake, body mass index, and number of long‐term medical conditions.

**Table 3:** Age and leucocyte telomere length (LTL) distribution between men and women across their frailty status

	Frailty status		
	Non‐frail	Pre‐frail	Frail
		(1–2 indicators)	(3–5 indicators)
*Women*			
*n* (%)	115 835 (48)	111 119 (46)	12 436 (5.2)
Age, years	56 (49; 62)	58 (51; 63)	59 (52; 64)
40 to 49, *n* (%)	30 965 (54)	23 783 (42)	2140 (3.8)
50 to 59, *n* (%)	41 018 (50)	37 231 (45)	4427 (5.4)
60 to 70, *n* (%)	43 852 (44)	50 105 (50)	5869 (5.9)
LTL, SD			
Overall	0.12 (0.98)	0.06 (0.99)	−0.03 (0.99)
40 to 49	0.32 (0.97)	0.31 (0.99)	0.26 (1.00)
50 to 59	0.16 (0.97)	0.13 (0.98)	0.02 (0.95)
60 to 70	−0.06 (0.97)	−0.10 (0.97)	−0.19 (0.99)
*Men*			
*n* (%)	107 813 (53)	86 496 (43)	8082 (4.0)
Age, years	57 (49; 63)	59 (51; 64)	60 (54; 65)
40 to 49, *n* (%)	26 964 (58)	18 571 (40)	1202 (2.6)
50 to 59, *n* (%)	35 797 (55)	26 488 (41)	2462 (3.8)
60 to 70, *n* (%)	45 052 (50)	41 437 (46)	4418 (4.9)
LTL, SD			
Overall	−0.07 (0.98)	−0.12 (0.99)	−0.28 (1.01)
40 to 49	0.22 (0.96)	0.21 (0.97)	0.11 (0.96)
50 to 59	−0.03 (0.96)	−0.05 (0.97)	−0.19 (0.97)
60 to 70	−0.27 (0.96)	−0.32 (0.97)	−0.43 (1.01)

Results shown as mean (SD) or median (1^st^ quartile; 3^rd^ quartile), unless otherwise indicated. Leucocyte telomere length (LTL) measurements are *z*‐standardized.

To assess the relationships between age, LTL, and frailty, a model with linear and quadratic terms for age and LTL was found to be the best model minimizing BIC (model M5, *Table* S2). In the presence of the quadratic terms, the age*LTL interaction was non‐significant (*Table* S2). Furthermore, the fitted values for the prevalence of non‐frailty, pre‐frailty, and frailty obtained from M5, for the average LTL (i.e. *z*‐LTL = 0SD), were similar to the observed frequencies with the small caveat that a crossover between the distributions of non‐frailty and pre‐frailty in the model occurs a year earlier (*Figure* S2).

Age was positively associated with being frail compared with being non‐frail with an average 3.4% higher risk per year of chronological age (M5; *Table* S2). Similarly, shorter LTL was positively associated with higher risk of frailty (11.5% higher risk for one SD shorter LTL), with the rate of change in the association of LTL with frailty dependent on the length of the telomere and modestly greater for shorter telomeres (*P* for quadratic term = 0.001) (*Table* S2). There were analogous associations of age and LTL with pre‐frailty but weaker than with frailty (*Table* S2). The coefficients for age and LTL from M5 were also similar to the ones derived from a generalized ordinal model (*Table* S3).

**Figure 1:**
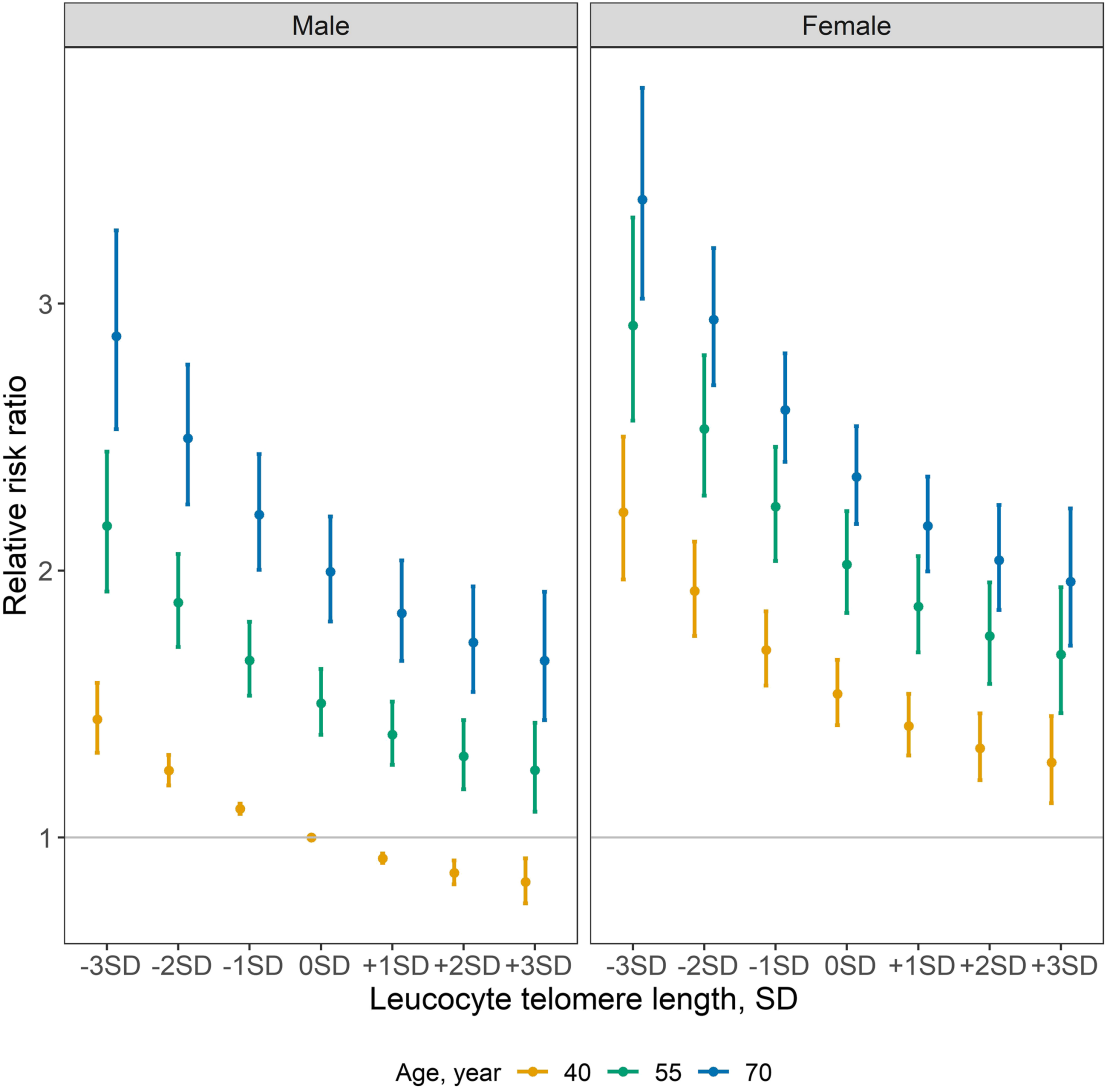
Relative risk ratios for the association of standardized leucocyte telomere length (LTL) with frailty, by age and sex. Relative risk ratios derived from the estimates of age and LTL for the frailty versus non‐frailty model shown in *Table* 4, compared with a 40 year old male while holding all the rest of the covariates constant. The gradient in the association between age and LTL is shown, with a slightly sharper decline for participants with LTL < 0SD compared with > 0SD (*P* for LTL quadratic term = 0.03) in both men and women. Compared with men, women have a higher risk of frailty in any given age or LTL group. Within sex, the age differences are more evident in men (*P* for interaction <0.0001).

We next evaluated whether sex (allowing for an interaction with age, *Table* S4) or the presence of other factors associated with frailty shown in *Table* 1, impacted on the effects of age and LTL on risk of frailty. Adjustment for these factors did not substantially alter the association of age with frailty (3.2% higher risk per year of chronological age, *Table* 4). Similarly, the association of shorter LTL with pre‐frailty [2.1% (1.4%; 2.7%) higher relative risk per one SD shorter LTL] and frailty [9.6% (7.9%; 11%) higher relative risk per one SD shorter LTL] remained highly significant after adjustment for these factors, with the rate of change of the association again modestly dependent on the length of the telomere (*P* for LTL quadratic term: 0.003 for pre‐frailty and 0.03 for frailty) (*Table* 4). The relative risk of frailty with different LTLs in men and women at age 40, 55, and 70, standardized to a 40 year old male, is shown in *Figure* 1. Across the spectrum of possible situations, there was a greater than three‐fold difference in the relative risk of frailty associated with variation in LTL. In a sensitivity analysis, to exclude the possibility that results are biased due to extreme telomere length, we revised the analysis for those within the −3SD to +3SD range of telomere length (*n* = 439 420 with complete data). Results remained consistent even after removing 2612 participants with extreme values in telomere length (*Table* S5).

**Table 4:** Adjusted relative risk ratios (RRR) and 95% confidence intervals (95% CI) from a multinomial logit model on frailty.

	Pre‐frail vs. non‐frail		Frail vs. non‐frail	
	RRR (95% CI)	*P*	RRR (95% CI)	*P*
Age, per year	0.992 (0.989; 0.995)	<0.0001	1.032 (1.023; 1.041)	<0.0001
Age^2	1.001 (1.001; 1.001)	<0.0001	1 (0.999; 1)	0.03
Telomere length, per SD shorter	1.021 (1.014; 1.027)	<0.0001	1.096 (1.079; 1.113)	<0.0001
Telomere length^2	1.006 (1.002; 1.01)	0.003	1.010 (1.001; 1.02)	0.03
Women vs. men	1.11 (1.079; 1.142)	<0.0001	1.538 (1.42; 1.665)	<0.0001
Age*Women	1.004 (1.003; 1.006)	<0.0001	0.991 (0.987; 0.995)	<0.0001
Fifths of deprivation*				
2nd vs. 1st (least deprived)	1.1 (1.082; 1.119)	<0.0001	1.306 (1.244; 1.371)	<0.0001
3rd vs. 1st	1.206 (1.184; 1.229)	<0.0001	1.658 (1.578; 1.741)	<0.0001
4th vs. 1st	1.352 (1.326; 1.379)	<0.0001	2.322 (2.215; 2.434)	<0.0001
5th (most deprived) vs. 1st	1.648 (1.612; 1.685)	<0.0001	3.645 (3.477; 3.821)	<0.0001
Smoking				
Previous vs. never	1.022 (1.008; 1.036)	0.002	1.098 (1.06; 1.136)	<0.0001
Current vs. never	1.398 (1.367; 1.428)	<0.0001	2.598 (2.486; 2.715)	<0.0001
Frequency of alcohol intake				
Daily vs. 1–4 times/week	0.896 (0.881; 0.911)	<0.0001	0.793 (0.755; 0.833)	<0.0001
1–3 times/month vs. 1–4 times/week	1.219 (1.195; 1.245)	<0.0001	1.522 (1.447; 1.601)	<0.0001
Occasionally/never vs. 1–4 times/week	1.556 (1.529; 1.584)	<0.0001	3.238 (3.121; 3.359)	<0.0001
Categories of body mass index				
<18.5 kg/m^2^ vs. 18.5–24.9 kg/m^2^	1.116 (1.021; 1.22)	0.02	1.874 (1.563; 2.248)	<0.0001
25–29.9 kg/m^2^ vs. 18.5–24.9 kg/m^2^	1.122 (1.106; 1.139)	<0.0001	1.163 (1.116; 1.213)	<0.0001
≥30 kg/m^2^ vs. 18.5–24.9 kg/m^2^	1.526 (1.5; 1.553)	<0.0001	2.607 (2.502; 2.716)	<0.0001
Number of co‐morbidities				
One LTC vs. none	1.219 (1.198; 1.24)	<0.0001	1.856 (1.735; 1.986)	<0.0001
Two LTCs vs. none	1.422 (1.396; 1.449)	<0.0001	3.207 (3.002; 3.426)	<0.0001
Three LTCs vs. none	1.66 (1.624; 1.697)	<0.0001	5.25 (4.91; 5.614)	<0.0001
Four or more LTCs vs. none	2.148 (2.102; 2.195)	<0.0001	12.51 (11.75; 13.31)	<0.0001

BIC = 699 491; pseudo‐*R* ^2^ = 6.6%.

* Fifths of deprivation were derived from the Townsend index of deprivation (2011). LTC: long‐term medical condition.

Correcting for regression dilution bias further strengthened the associations of LTL with both frailty conditions [3.1% (2.1%; 4.0%) and 14% (12%; 17%) higher, for pre‐frailty and frailty respectively per one SD shorter LTL]. Adjustment for the associations of LTL with 123 prevalent diseases reduced the associations of LTL with both pre‐frailty and frailty but both remained significant [1.4% (0.8%; 2.1%) and 4.5% (2.9%; 6.1%) higher per one SD shorter LTL, *P* < 0.0001 for both].

In the full model, sex and other established risk factors were substantially associated with both pre‐frailty and frailty (*Table* 4). For example, compared to men, women had about 50% higher relative risk of being frail as opposed to being non‐frail, while being in the highest fifth of social deprivation involved about 3.5‐fold higher risk than being in the lowest fifth. Each additional LTC was associated with sharply higher risk of frailty. In particular, participants with at least four LTCs had about 12.5‐fold higher risk of frailty compared with those with no LTC (*Table* 4). Overall, the variables we analysed explained approximately 6.6% of the variance in the distribution of the frailty phenotypes.

**Figure 2:**
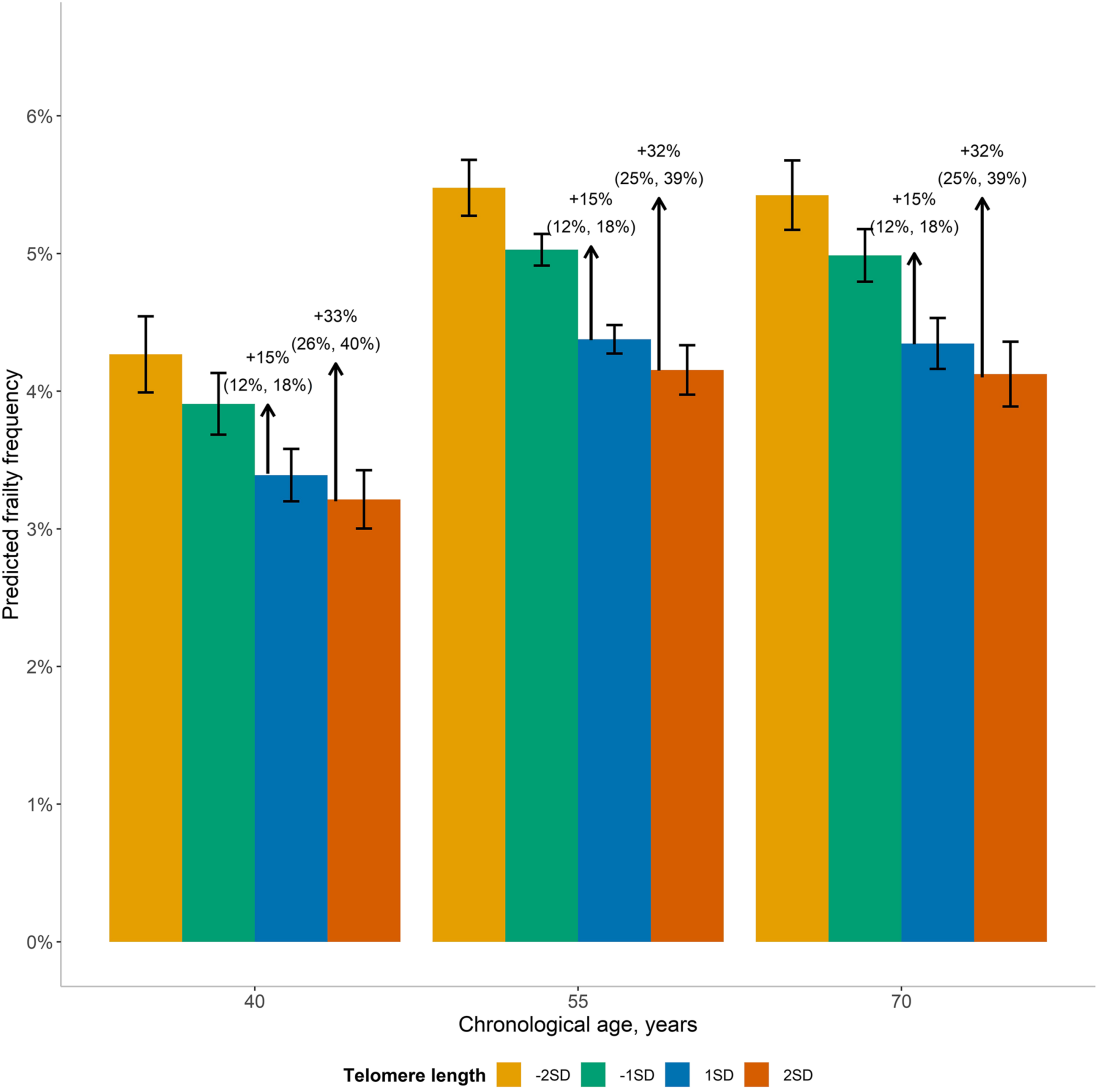
Predicted frequencies of frailty by chronological age, over four specific telomere lengths. Predicted frequency of frailty is derived from the estimates of age and leucocyte telomere length (LTL) for the frailty versus non‐frailty model shown in *Table* 4, holding all the rest of the covariates at their observed values. Bars indicate the average predicted frequency of frailty, while error bars indicate the 95% confidence interval (CI). The ratio between two predicted frequencies (95% CI) is also given. There are approximately 2% (8364) participants with LTL equal to −2SD or +2SD, and 9% (41 768) participants with LTL equal to −1SD or +1SD.

The predicted absolute frequency of frailty, derived from the full model, across the population distribution of age, stratified by LTL and accounting for other risk factors, is shown in *Figure* 2. In each age group, there was a gradient in increased frequency of frailty moving from longer to shorter LTL without any threshold effect. The strength of the association between LTL and frailty appeared similar in each of the different age categories. Thus, in each age group, the frequency of frailty was about 15% higher in participants with LTL one SD shorter versus one SD longer than the mean and about 33% higher between two SD either side of the mean (*Figure* 2).

**Figure 3:**
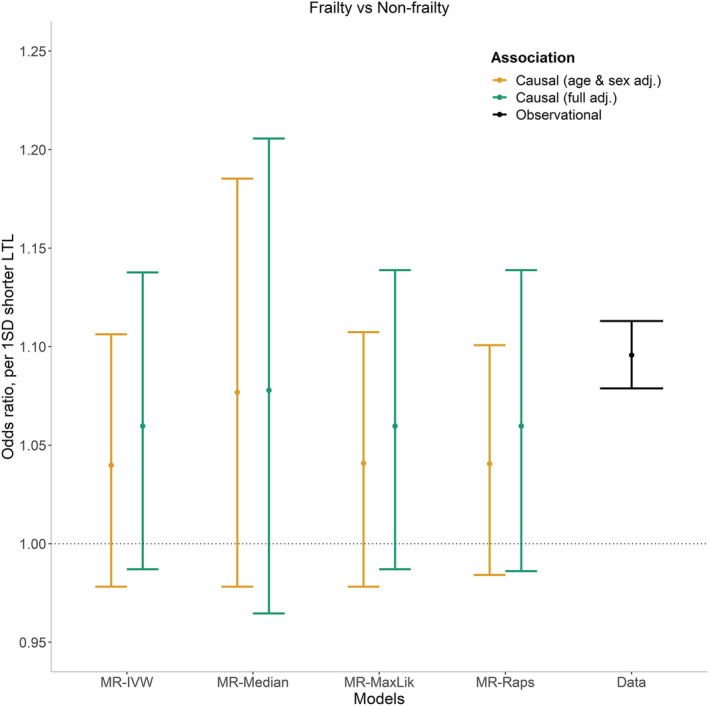
Results from Mendelian randomization (MR) and multinomial regression to assess respectively the causal (*MR*) and the observational (*data*) association between telomere length and frailty. In addition to array and first 10 principal components of the age & sex adjusted causal model, covariates included in the fully adjusted causal model were deprivation, alcohol intake, smoking, body mass index, and number of long‐term medical conditions. Point estimates represent odds ratios, bars represent 95% confidence intervals.

In the causal inference analysis there was a similar trend towards an association between shorter LTL and greater risk of frailty with a point estimate that overlapped with the observational association (*Figure* 3). However, the 95% confidence intervals were wide and the association was not significant (OR 1.08 (0.98; 1.19) per one SD shorter genetically determined LTL (*P* = 0.13) from the MR‐Median model). Different MR approaches (see ‘Methods section’ in the supporting information) yielded similar results (*Figure* 3); in particular, there was no evidence of substantial pleiotropy (MR Egger intercept's *P*‐value = 0.60). Adjustment for additional covariates did not attenuate the trend (*Figure* 3), while we have found no evidence of a quadratic trend in the association between the genetically‐determined LTL and frailty (Quadratic *P* = 0.506).

## Discussion

Utilizing the powerful resource of the UK Biobank, in which we measured LTL in over 472 000 participants, we report a significant association between shorter LTL and greater risk of frailty in a contemporary population. We demonstrate that progressively shorter telomere lengths associate with non‐frailty through pre‐frailty to frailty across all age groups. The association between shorter LTL and greater risk of frailty remained significant after adjustment for other established determinants of frailty such as age, sex, alcohol intake, smoking, obesity, deprivation, and multimorbidity. Furthermore, the associations of shorter LTL with pre‐frailty and frailty were at least partly independent of the potential associations between variation in LTL and 123 prevalent diseases spanning multiple body systems.

A few studies have previously examined the relationship between frailty and telomere with variable findings. Two recent meta‐analyses of these studies, including up to 3268^14^ and 10 079^16^ individuals respectively, and a recent MR study,^24^ did not show a consistent association between shorter LTL and frailty indices. While several factors, including the age ranges and ethnicities studied, the method to estimate telomere length and the definition of frailty, may contribute to the heterogeneous findings, the most likely reason is statistical power. Our study analysed over 40‐fold more participants than the largest meta‐analysis providing greater power to detect any association between variation in LTL and frailty as well as pre‐frailty. Additionally, the genetic instrument we used consisted of 131 SNPs, while the one used by Kuo *et al*.^24^ utilized only 13 available SNPs, suggesting a difference in the power of the instruments. In producing our genetic instrument^17^ we removed potential pleiotropic variants and so therefore feel that this is a robust telomere‐specific instrument.

Fried's frailty phenotype^3^ integrates five different functional measures. We confirmed both an association of similar magnitudes of LTL with individual components of this phenotype and also, importantly, that the association is stronger as the number of frailty indicators increase. These findings indicate that the observed association is not due to one of the component phenotypes.

At a tissue level, LTL is a determinant of replicative capacity and tissue repair.^10^, ^13^ Thus, the association of shorter LTL with increased risk of frailty could be explained by earlier exhaustion of these functions across multiple body systems. However, a notable finding was that the *relative* increase in risk of frailty with shorter telomeres was similar in different age groups and did not increase with age. This suggests that LTL is not simply accelerating the effect of chronological age on risk of frailty as a biomarker of premature ageing. This is in accord with recent concepts around telomere dynamics and ageing‐related diseases.^13^ However, it should be noted that the age range at recruitment of participants in UK was relatively narrow at between 40–69 years and we cannot exclude the possibility that the relative association of shorter LTL with risk of frailty might be stronger at older ages. A further concern about UKB is that, because recruitment was voluntary and required participants to travel to a recruitment centre, it may have recruited relatively ‘healthy’ individuals particularly at older ages (60–69).^25^ This could impact on the prevalence of the frailty phenotype and hence the generalizability of the findings.^25^ However, we observed similar estimates for the age and sex‐specific rates of frailty syndrome at the overlapping age range available (60–75 years) with two other studies available in the UK: those derived from the Hertfordshire Cohort Study^26^ and from the English Longitudinal Study of Ageing.^27^ Furthermore, previous analysis has shown that even if the prevalence of a disorder is different in UKB compared with a general population, it should not impact on its relative association with a risk factor.^28^

While the association of shorter LTL with risk of frailty was highly significant and there was approximately 33% higher risk of frailty in those with two SD shorter LTL compared with those with two SD longer LTL than average, the association needs to be viewed in context of other risk factors for frailty. As shown in *Table* 4, socio‐demographic and lifestyle factors such as social deprivation, BMI, alcohol intake, smoking and presence of co‐morbidity individually all had much more powerful associations than LTL.

Although the richness of the information on participants in UKB allowed us to adjust for several relevant factors in assessing the association of LTL with risk of frailty, a cross‐sectional analysis cannot infer causation. To investigate whether the association of LTL with risk if frailty was causal, we deployed Mendelian randomization using 131 genetic variants associated with LTL^12^ as instruments. Although this showed an association that was concordant with the observational finding both directionally and in terms of effect size, the 95% margins were wide, indicating limited power to confirm or exclude a genetic association. This probably reflects a combination of the low overall prevalence of frailty in the studied population and the relatively low strength of the genetic instruments, which explain less than 5% of the variation in LTL.^12^ Therefore, we cannot exclude the possibility that the observed association between shorter LTL and increased risk of frailty is due to residual confounding which we have not accounted for.

Telomere length is largely genetically determined.^12^, ^29^ However, several studies have shown that lifestyle factors including smoking, diet, physical activity and body mass index also associate with LTL.^30^, ^31^, ^32^, ^33^, ^34^ Furthermore, there is evidence from animal studies that restoration of telomere length can reverse age‐related phenotypes.^35^ Therefore, if the relationship between shorter LTL and increased risk of frailty can be confirmed to be causal, preservation of LTL through lifestyle changes or safe manipulation of telomere length may emerge as a novel target to reduce the risk of frailty.

More broadly, our analysis and findings have relevance to definitional approaches, which have been developed for the characterization of frailty.^2^, ^3^ The concept of frailty attempts to explain the heterogeneity in health and functional status, as individuals get older, which is thought to arise from a reduction in reserve capacity in various physiologic systems. In the present analysis, LTL‐frailty associations were at least partly independent of the presence of long‐term co‐morbidities, included either as number of conditions or via the residuals derived from LTL regressed on the wider set of 123 morbidities. This suggests that there is, in addition to ‘accumulated morbidity’, an element of the frailty syndrome that is independent of co‐morbidities, as proposed by Fried *et al*.,^3^ thereby supporting the notion that the syndromic approach may identify elements of vulnerability and resilience that distinguish frailty from disability or disease that accumulate over time.^1^, ^36^

Despite the scale of our study and the uniform and detailed phenotypic characterization in UKB, some limitations, in addition to those discussed earlier, should be considered in the interpretation of our findings. First, as information to derive the frailty phenotype was only collected at baseline, we are unable to investigate any relationship of inter‐individual variation in LTL to future development of frailty. Similarly, our single point estimate of LTL precludes analysis of the association of any changes in LTL with age and development of frailty. Finally, UKB predominantly comprises individuals of white ethnicity. There are differences in average LTL (adjusting for age and gender) in participants from different ethnicities^17^ and whether the association of LTL with risk of frailty differs in participants from different backgrounds remains to be investigated.

In summary, we show that shorter LTL is associated with greater risk of syndromic frailty and that this association is independent of other risk factors but partly explained by the causal association of LTL with diseases across multiple body systems. Our findings provide evidence for an additional biological factor associated with the risk of frailty.

## Conflict of interest

All authors declare no conflict of interest.

## Funding

This research has been conducted using the UK Biobank Resource under Application Number 6077. Generation of the LTL measurements was funded by the UK Medical Research Council (MRC), Biotechnology and Biological Sciences Research Council and British Heart Foundation (BHF) through MRC grant MR/M012816/1. The funders had no role in study design; the collection, analysis, and interpretation of data; the writing of the report; and the decision to submit the paper for publication.

## Supporting information




**Table S1:** Adjusted relative risk ratios (RRR) and 95% confidence intervals (95% CI) from a multinomial regression model^∫^ of age, sex and leucocyte telomere length on number of frailty indicators.
**Table S2:** Relative risk ratios (RRR) and 95% confidence intervals (95% CI) from multinomial logit models of age and leucocyte telomere length on frailty.
**Table S3:** Adjusted odds ratios (OR) and 95% confidence intervals (95% CI) from a generalized ordinal model of age and leucocyte telomere length on frailty.
**Table S4:** Adjusted relative risk ratios (RRR) and 95% confidence intervals (95% CI) from multinomial logit models of age, leucocyte telomere length (LTL) and sex on frailty.
**Table S5:** Adjusted relative risk ratios (RRR) and 95% confidence intervals (95% CI) from a multinomial logit model on frailty, excluding *n* = 2,612 UKB participants with telomere length beyond ±3SDs (*n* = 439,420 with complete data).
**Figure S1:** Flow of participants in the study.
**Figure S2:** Participants' distribution by frailty status over age, as occurred from the observed data and the fitted values from model M5 (~age+age^2^ + LTL + LTL^2^), for average LTL (0SD).
