# Supplementary material for: Association of shorter leucocyte telomere length with risk of frailty
Source: J Cachexia Sarcopenia Muscle. 2022 Mar 17;13(3):1741–51. doi: 10.1002/jcsm.12971 (PMC9178164; doi:10.1002/jcsm.12971)

**Association of shorter leucocyte telomere length with risk of frailty: Online supplementary material**

**Methods**

**Statistical analysis**

Seven sequential multinomial logistic regression models were initially tested: i) a univariable model including only age (M1a), ii) a univariable model including only leucocyte telomere length (LTL; M1b), iii) a model including age, LTL and their interaction (M2), iv) M2 with an additional quadratic term for age (M3a), v) M2 with an additional quadratic term for LTL (M3b), vi) a model with age, LTL, their interaction and their quadratic terms (M4), and vii) a model with age, LTL and their quadratic terms (M5). Models were compared using the Bayesian information criterion (BIC), and the model with the lowest BIC was selected.

Following the best model selection for age and LTL (M5), a model including sex (females/ males) as an additional predictor and further models with interaction terms of sex with age and LTL were also evaluated. Finally, the best selected model from this step (M5+sex+age*sex) was additionally adjusted for other well–known determinants of frailty (see main paper).

In a sensitivity analysis we also performed a generalised ordinal model [1], loosening the assumption of proportionality, with the frailty phenotype (none/ pre–frail/ frail) as an outcome. In addition, we performed binary logistic regression models to investigate the association between LTL and the individual frailty indicators, as well a multinomial logistic regression model for the association between LTL and the total number of frailty indicators.

To explore whether LTL is causally associated with the phenotype of frailty we performed a genetic analysis using 131 genetic variants associated with LTL [2] as instrumental variables. To estimate the genetic associations of the variants with z–standardised LTL (beta1) we conducted linear regression analysis between the z–standardised LTL and each genetic variant while adjusting for age (centred at the age of 40), sex, array, and the first 10 principal components as described previously [2]. To estimate the associations of genetic variants with frailty (beta2) we performed two different logistic regression analyses between frailty and each genetic variant in turn. In the first analysis we adjusted the model only for the same covariates listed above, whilst in the second analysis we additionally adjusted the model deprivation, alcohol intake, smoking, BMI and LCTs, as these were significantly associated with the prevalence of frailty using the observational data. The causal association between LTL and the phenotype of frailty was assessed through one–sample Mendelian Randomization (MR) [3], utilising different methods to test for robustness of findings. In particular, to estimate the odds ratio for frailty per one SD shorter LTL we used four different MR functions: i) the inverse variance weighted MR (IVW), ii) the median based estimator (Median) and iii) the maximum likelihood (MaxLik) of the “MendelianRandomisation” package [4], and iv) the Robust Adjusted Profile (RAPS) of the “mr.raps” package [5]. The selected methods complement each other in the assumptions that should be satisfied in order to produce valid results and were used in parallel. Particularly, the IVW is the most efficient and robust method in the absence of pleiotropy, the Median is additionally robust in the presence of outliers, the MaxLik allows a) for uncertainty in the genetic associations with the exposure and b) for genetic associations with the exposure and with the outcome for each variant to be correlated and the RAPS method overcomes challenges related to measurement error, weak or invalid (due to pleiotropy) measurements and selection bias (due to weak instrument). Therefore a combination of these methods provides the best evidence for the presence of a causal association [6, 7]. In addition, the presence of pleiotropy was assessed through the MR–Egger analysis [8]. **To examine whether a non-linear association of the genetic effect of telomere length with frailty was also present we performed a non-linear MR using the “jrs95/nlmr” package [9]. In brief, the non-linear MR incorporates the concept of instrumental variables (IV) to estimate the causal effect of an exposure to an outcome, using the genetic variants as IV [10]. For each participant we computed a weighted allele score (i.e. genetic risk score) by multiplying the number of effect alleles associated with telomere length for each variant by the effect of the variant on telomere length and summing across the 131 variants. This weighted allele score was the IV used in the non-linear MR.**

MR analyses were performed in R version 3.1.6 [**11**], whilst the regression models were performed in Stata v16.0 [**12**]. Figures were produced using the “ggplot2” package in R [**13**]. The significance level for all associations was set at 0.05.

**References**

1. Williams R. Understanding and interpreting generalized ordered logit models. *J Math Sociol.* 2016; **40**: 7–20.
2. Codd V, Wang C, Allara E, et al. Polygenic basis and biomedical consequences of telomere length variation. medRxiv 2021.03.23.21253516; doi: https://doi.org/10.1101/2021.03.23.21253516.
3. Broadbent JR, Foley CN, Grant AJ et al. MendelianRandomization v0.5.0: updates to an R package for performing Mendelian randomization analyses using summarized data [version 2; peer review: 1 approved, 2 approved with reservations]. *Wellcome Open Res.* 2020; **5**: 252.
4. Yavorska O, Staley J. MendelianRandomization: Mendelian Randomization Package. 2020. R package version 0.5.0.
5. Zhao O, Wang J, Bowden J, Small DS. Statistical inference in two–sample summary–data Mendelian randomization using robust adjusted profile score. *Ann Statist*. 2020: **48**: 1742–1769.
6. Burgess S, Butterworth A, Thompson SG. Mendelian randomization analysis with multiple genetic variants using summarized data. *Genet Epidemiol.* 2013; **37**: 658–665.
7. Bowden J, Davey Smith G, Burgess S. Mendelian randomization with invalid instruments: effect estimation and bias detection through Egger regression. *Int J Epidemiol.* 2015; **44**: 512–525.
8. Bowden J, Davey Smith G, Haycock PC, Burgess S. Consistent Estimation in Mendelian Randomization with Some Invalid Instruments Using a Weighted Median Estimator. *Genet Epidemiol.* 2016; **40**: 304–314.
9. **Staley J. nlmr: Non-linear Mendelian randomisation. R package version 2.0.**
10. **Staley JR, Burgess S. Semiparametric methods for estimation of a non-linear exposure-outcome relationship using instrumental variables with application to Mendelian randomization. *Genet Epidemiol.* 2017; 41: 341–352.**
11. R Core Team. R: A language and environment for statistical computing. Version 3.1.6 (Action of the Toes). R Foundation for Statistical Computing, Vienna, Austria, 2019.
12. StataCorp. 2019. Stata Statistical Software: Release 16. College Station, TX: StataCorp LLC.
13. Wickham H. ggplot2: Elegant Graphics for Data Analysis. Springer-Verlag New York, 2016.

**Supplementary Table 1: Adjusted relative risk ratios (RRR) and 95% confidence intervals (95% CI) from a multinomial regression model^∫^ of age, sex and leucocyte telomere length on number of frailty indicators.**

|  | Number of frailty indicators | | | | | | | | | | | |
| --- | --- | --- | --- | --- | --- | --- | --- | --- | --- | --- | --- | --- |
|  | 1 vs 0 | | 2 vs 0 | | 3 vs 0 | | 4 vs 0 | | | 5 vs 0 | | |
|  | RRR (95% CI) | *P* | RRR (95% CI) | *P* | RRR (95% CI) | *P* | | RRR (95% CI) | *P* | | RRR (95% CI) | *P* |
| Age, per year increase | 0.99 | <0.0001 | 1.004 | 0.17 | 1.03 | <0.0001 | | 1.04 | <0.0001 | | 1.11 | <0.0001 |
|  | (0.985; 0.992) |  | (0.998; 1.009) |  | (1.02; 1.04) |  | | (1.02; 1.06) |  | | (1.06; 1.17) |  |
| Age^2 | 1.001 | <0.0001 | 1 | <0.0001 | 1 | 0.34 | | 0.999 | 0.03 | | 0.998 | 0.002 |
|  | (1.001; 1.001) |  | (1; 1.001) |  | (1; 1) |  | | (0.999; 1) |  | | (0.996; 0.999) |  |
| Telomere length, per SD shorter | 1.01 | <0.0001 | 1.04 | <0.0001 | 1.09 | <0.0001 | | 1.12 | <0.0001 | | 1.21 | <0.0001 |
|  | (1.007; 1.02) |  | (1.03; 1.05) |  | (1.07; 1.11) |  | | (1.09; 1.16) |  | | (1.11; 1.32) |  |
| Telomere length^2 | 1.002 | 0.29 | 1.02 | <0.0001 | 1.02 | 0.003 | | 1 | 0.98 | | 0.97 | 0.28 |
|  | (0.998; 1.01) |  | (1.011; 1.02) |  | (1.005; 1.03) |  | | (0.981; 1.02) |  | | (0.92; 1.02) |  |
| Females vs Males | 1.07 | <0.0001 | 1.27 | <0.0001 | 1.54 | <0.0001 | | 1.61 | <0.0001 | | 2.03 | 0.003 |
|  | (1.04; 1.1) |  | (1.21; 1.34) |  | (1.4; 1.68) |  | | (1.36; 1.9) |  | | (1.28; 3.21) |  |
| Age*Females | 1.005 | <0.0001 | 1 | 0.86 | 0.993 | 0.001 | | 0.986 | 0.001 | | 0.972 | 0.02 |
|  | (1.003; 1.007) |  | (0.998; 1.003) |  | (0.988; 0.997) |  | | (0.978; 0.995) |  | | (0.95; 0.995) |  |

^∫^Model additionally adjusted for fifths of Townsend index of deprivation (2011), smoking, alcohol intake, body mass index and number of long–term medical conditions.

**Supplementary Table 2: Relative risk ratios (RRR) and 95% confidence intervals (95% CI) from multinomial logit models of age and leucocyte telomere length on frailty.**

|  | BIC; | Pre–frail *vs.* Non–frail | | Frail *vs.* Non–frail | |
| --- | --- | --- | --- | --- | --- |
|  | pseudo–R^2^ | RRR (95% CI) | *P* | RRR (95% CI) | *P* |
| *M1a* | 745093; |  |  |  |  |
|  | 0.45% |  |  |  |  |
| Age, per year |  | 1.019 | <0.0001 | 1.037 | <0.0001 |
|  |  | (1.018; 1.02) |  | (1.035; 1.039) |  |
| *M1b* | 747826; |  |  |  |  |
|  | 0.09% |  |  |  |  |
| Telomere length, per SD shorter |  | 1.051 | <0.0001 | 1.178 | <0.0001 |
|  |  | (1.045; 1.058) |  | (1.161; 1.195) |  |
| *M2* | 744886; |  |  |  |  |
|  | 0.49% |  |  |  |  |
| Age, per year |  | 1.019 | <0.0001 | 1.034 | <0.0001 |
|  |  | (1.018; 1.02) |  | (1.032; 1.036) |  |
| Telomere length, per SD shorter |  | 0.988 | 0.09 | 1.083 | <0.0001 |
|  |  | (0.974; 1.002) |  | (1.044; 1.124) |  |
| Age*Telomere length |  | 1.002 | <0.0001 | 1.002 | 0.05 |
|  |  | (1.001; 1.003) |  | (1; 1.004) |  |
| *M3a* | 744663; |  |  |  |  |
|  | 0.52% |  |  |  |  |
| Age, per year |  | 0.994 | 0.001 | 1.035 | <0.0001 |
|  |  | (0.991; 0.998) |  | (1.027; 1.044) |  |
| Age^2 |  | 1.001 | <0.0001 | 1 | 0.98 |
|  |  | (1.001; 1.001) |  | (1; 1) |  |
| Telomere length, per SD shorter |  | 1.007 | 0.36 | 1.081 | <0.0001 |
|  |  | (0.992; 1.021) |  | (1.041; 1.122) |  |
| Age*Telomere length |  | 1.001 | 0.04 | 1.002 | 0.05 |
|  |  | (1; 1.002) |  | (1; 1.004) |  |
| *M3b* | 744894; |  |  |  |  |
|  | 0.49% |  |  |  |  |
| Age, per year |  | 1.019 | <0.0001 | 1.034 | <0.0001 |
|  |  | (1.018; 1.020) |  | (1.032; 1.036) |  |
| Telomere length, per SD shorter |  | 0.993 | 0.37 | 1.094 | <0.0001 |
|  |  | (0.979; 1.008) |  | (1.054; 1.134) |  |
| Telomere length^2 |  | 1.002 | 0.001 | 1.001 | 0.002 |
|  |  | (1.001; 1.002) |  | (0.999; 1.003) |  |
| Age*Telomere length |  | 1.007 | <0.0001 | 1.015 | 0.23 |
|  |  | (1.003; 1.011) |  | (1.005; 1.024) |  |
| *M4* | 744669; |  |  |  |  |
|  | 0.52% |  |  |  |  |
| Age, per year |  | 0.994 | <0.0001 | 1.035 | <0.0001 |
|  |  | (0.991; 0.997) |  | (1.027; 1.043) |  |
| Age^2 |  | 1.001 | <0.0001 | 1 | 0.97 |
|  |  | (1.001; 1.001) |  | (1; 1) |  |
| Telomere length, per SD shorter |  | 1.013 | 0.08 | 1.092 | <0.0001 |
|  |  | (0.998; 1.028) |  | (1.051; 1.134) |  |
| Telomere length^2 |  | 1.008 | <0.0001 | 1.015 | 0.002 |
|  |  | (1.004; 1.012) |  | (1.005; 1.024) |  |
| Age*Telomere length |  | 1 | 0.27 | 1.001 | 0.23 |
|  |  | (1; 1.001) |  | (0.999; 1.003) |  |
| ***M5*** | 744645; |  |  |  |  |
|  | 0.52% |  |  |  |  |
| Age, per year |  | 0.994 | <0.0001 | 1.034 | <0.0001 |
|  |  | (0.991; 0.997) |  | (1.026; 1.042) |  |
| Age^2 |  | 1.001 | <0.0001 | 1 | 0.78 |
|  |  | (1.001; 1.001) |  | (1; 1) |  |
| Telomere length, per SD shorter |  | 1.02 | <0.0001 | 1.115 | <0.0001 |
|  |  | (1.014; 1.027) |  | (1.098; 1.131) |  |
| Telomere length^2 |  | 1.008 | <0.0001 | 1.016 | <0.0001 |
|  |  | (1.004; 1.012) |  | (1.007; 1.025) |  |

The model in **bold** indicates the best model selected on the basis of minimizing the BIC.

**Supplementary Table 3: Adjusted odds ratios (OR) and 95% confidence intervals (95% CI) from a generalised ordinal model of age and leucocyte telomere length on frailty.**

|  | Pre–/ Frail *vs.* Non–frail | | Frail *vs.* Pre– and Non–frail | |
| --- | --- | --- | --- | --- |
|  | OR (95% CI) | *P* | OR (95% CI) | *P* |
| *M5* |  |  |  |  |
| Age, per year | 0.997 | 0.05 | 1.039 | <0.0001 |
|  | (0.994; 1.000) |  | (1.031; 1.047) |  |
| Age^2 | 1.001 | <0.0001 | 1 | 0.001 |
|  | (1.001; 1.001) |  | (0.999; 1) |  |
| Telomere length, per SD shorter | 1.029 | <0.0001 | 1.104 | <0.0001 |
|  | (1.023; 1.035) |  | (1.089; 1.12) |  |
| Telomere length^2 | 1.010 | <0.0001 | 1.010 | <0.0001 |
|  | (1.006; 1.013) |  | (1.006; 1.013) |  |

BIC=744629; pseudo–R^2^=0.52%.

**Supplementary Table 4: Adjusted relative risk ratios (RRR) and 95% confidence intervals (95% CI) from multinomial logit models of age, leucocyte telomere length (LTL) and sex on frailty.**

|  | BIC; | Pre–frail vs Non–frail | | Frail vs Non–frail | |
| --- | --- | --- | --- | --- | --- |
|  | pseudo–R^2^ | RRR (95% CI) | *P* | RRR (95% CI) | *P* |
| *M5+sex* | 743217; |  |  |  |  |
|  | 0.72% |  |  |  |  |
| Age, per year |  | 0.993 | <0.0001 | 1.031 | <0.0001 |
|  |  | (0.989; 0.996) |  | (1.023; 1.039) |  |
| Age^2 |  | 1.001 | <0.0001 | 1 | 0.26 |
|  |  | (1.001; 1.001) |  | (1; 1) |  |
| Telomere length, per SD shorter |  | 1.030 | <0.0001 | 1.136 | <0.0001 |
|  |  | (1.023; 1.036) |  | (1.119; 1.153) |  |
| Telomere length^2 |  | 1.008 | <0.0001 | 1.016 | 0.001 |
|  |  | (1.004; 1.012) |  | (1.007; 1.025) |  |
| Female vs male |  | 1.219 | <0.0001 | 1.499 | <0.0001 |
|  |  | (1.204; 1.234) |  | (1.455; 1.543) |  |
| ***M5+sex+age*sex*** | 743168; |  |  |  |  |
|  | 0.73% |  |  |  |  |
| Age, per year |  | 0.989 | <0.0001 | 1.034 | <0.0001 |
|  |  | (0.986; 0.992) |  | (1.026; 1.043) |  |
| Age^2 |  | 1.001 | <0.0001 | 1 | 0.29 |
|  |  | (1.001; 1.001) |  | (1; 1) |  |
| Telomere length, per SD shorter |  | 1.030 | <0.0001 | 1.136 | <0.0001 |
|  |  | (1.024; 1.037) |  | (1.119; 1.153) |  |
| Telomere length^2 |  | 1.009 | <0.0001 | 1.016 | 0.001 |
|  |  | (1.005; 1.012) |  | (1.007; 1.024) |  |
| Females vs Males |  | 1.103 | <0.0001 | 1.629 | <0.0001 |
|  |  | (1.072; 1.134) |  | (1.511; 1.757) |  |
| Age*Females |  | 1.006 | <0.0001 | 0.996 | 0.03 |
|  |  | (1.005; 1.008) |  | (0.992; 1.000) |  |
| *M5+sex+age*sex+LTL*sex* | 743188; |  |  |  |  |
|  | 0.73% |  |  |  |  |
| Age, per year |  | 0.989 | <0.0001 | 1.034 | <0.0001 |
|  |  | (0.986; 0.992) |  | (1.025; 1.042) |  |
| Age^2 |  | 1.001 | <0.0001 | 1 | 0.28 |
|  |  | (1.001; 1.001) |  | (1; 1) |  |
| Telomere length, per SD shorter |  | 1.027 | <0.0001 | 1.158 | <0.0001 |
|  |  | (1.018; 1.037) |  | (1.131; 1.186) |  |
| Telomere length^2 |  | 1.009 | <0.0001 | 1.014 | 0.002 |
|  |  | (1.005; 1.013) |  | (1.005; 1.023) |  |
| Females vs Males |  | 1.105 | <0.0001 | 1.615 | <0.0001 |
|  |  | (1.074; 1.136) |  | (1.497; 1.743) |  |
| Age*Females |  | 1.006 | <0.0001 | 0.997 | 0.08 |
|  |  | (1.004; 1.007) |  | (0.993; 1) |  |
| Telomere length*Females |  | 1.006 | 0.38 | 0.969 | 0.04 |
|  |  | (0.993; 1.019) |  | (0.94; 0.999) |  |

The model in **bold** indicates the best model selected on the basis of minimizing the BIC.

**Supplementary Table 5: Adjusted relative risk ratios (RRR) and 95% confidence intervals (95% CI) from a multinomial logit model on frailty, excluding n=2,612 UKB participants with telomere length beyond ±3SDs (n=439,420 with complete data).**

|  | Pre–frail vs Non–frail | | Frail vs Non–frail | |
| --- | --- | --- | --- | --- |
|  | RRR (95% CI) | *P* | RRR (95% CI) | *P* |
| Age, per year | 0.992 | <0.0001 | 1.032 | <0.0001 |
|  | (0.989; 0.995) |  | (1.023; 1.041) |  |
| Age^2 | 1.001 | <0.0001 | 1 | 0.029 |
|  | (1.001; 1.001) |  | (0.999; 1) |  |
| Telomere length, per SD shorter | 1.022 | <0.0001 | 1.102 | <0.0001 |
|  | (1.016; 1.029) |  | (1.084; 1.12) |  |
| Telomere length^2 | 1.005 | 0.035 | 1.007 | 0.225 |
|  | (1; 1.01) |  | (0.996; 1.019) |  |
| Females vs Males | 1.109 | <0.0001 | 1.528 | <0.0001 |
|  | (1.078; 1.141) |  | (1.411; 1.655) |  |
| Age*Females | 1.004 | <0.0001 | 0.991 | <0.0001 |
|  | (1.003; 1.006) |  | (0.987; 0.995) |  |
| Fifths of deprivation* |  |  |  |  |
| 2nd vs 1st (least deprived) | 1.1 | <0.0001 | 1.304 | <0.0001 |
|  | (1.082; 1.119) |  | (1.243; 1.369) |  |
| 3rd vs 1st | 1.206 | <0.0001 | 1.659 | <0.0001 |
|  | (1.183; 1.228) |  | (1.58; 1.743) |  |
| 4th vs 1st | 1.353 | <0.0001 | 2.325 | <0.0001 |
|  | (1.327; 1.379) |  | (2.218; 2.437) |  |
| 5ht (most deprived) vs 1st | 1.648 | <0.0001 | 3.655 | <0.0001 |
|  | (1.612; 1.685) |  | (3.486; 3.832) |  |
| Smoking |  |  |  |  |
| Previous vs never | 1.023 | 0.001 | 1.098 | <0.0001 |
|  | (1.009; 1.037) |  | (1.061; 1.137) |  |
| Current vs never | 1.398 | <0.0001 | 2.595 | <0.0001 |
|  | (1.368; 1.429) |  | (2.483; 2.712) |  |
| Frequency of alcohol intake |  |  |  |  |
| Daily vs 1–4 times/week | 0.896 | <0.0001 | 0.79 | <0.0001 |
|  | (0.881; 0.911) |  | (0.752; 0.829) |  |
| 1–3 times/month vs 1–4 times/week | 1.22 | <0.0001 | 1.52 | <0.0001 |
|  | (1.195; 1.245) |  | (1.445; 1.599) |  |
| Occasionally/ never vs 1–4 times/week | 1.555 | <0.0001 | 3.233 | <0.0001 |
|  | (1.528; 1.583) |  | (3.116; 3.354) |  |
| Categories of body mass index |  |  |  |  |
| <18.5 kg/m^2^ vs 18.5–24.9 kg/m^2^ | 1.11 | 0.022 | 1.87 | <0.0001 |
|  | (1.015; 1.214) |  | (1.558; 2.244) |  |
| 25–29.9 kg/m^2^ vs 18.5–24.9 kg/m^2^ | 1.122 | <0.0001 | 1.16 | <0.0001 |
|  | (1.106; 1.139) |  | (1.113; 1.21) |  |
| ≥30 kg/m^2^ vs 18.5–24.9 kg/m^2^ | 1.526 | <0.0001 | 2.603 | <0.0001 |
|  | (1.5; 1.553) |  | (2.498; 2.712) |  |
| Number of comorbidities |  |  |  |  |
| One LTC vs none | 1.22 | <0.0001 | 1.861 | <0.0001 |
|  | (1.199; 1.241) |  | (1.739; 1.992) |  |
| Two LTCs vs none | 1.423 | <0.0001 | 3.217 | <0.0001 |
|  | (1.396; 1.45) |  | (3.011; 3.437) |  |
| Three LTCs vs none | 1.66 | <0.0001 | 5.267 | <0.0001 |
|  | (1.624; 1.697) |  | (4.925; 5.633) |  |
| Four or more LTCs vs none | 2.148 | <0.0001 | 12.56 | <0.0001 |
|  | (2.102; 2.195) |  | (11.80; 13.37) |  |

**Supplementary Figure 1: Flow of participants in the study.**


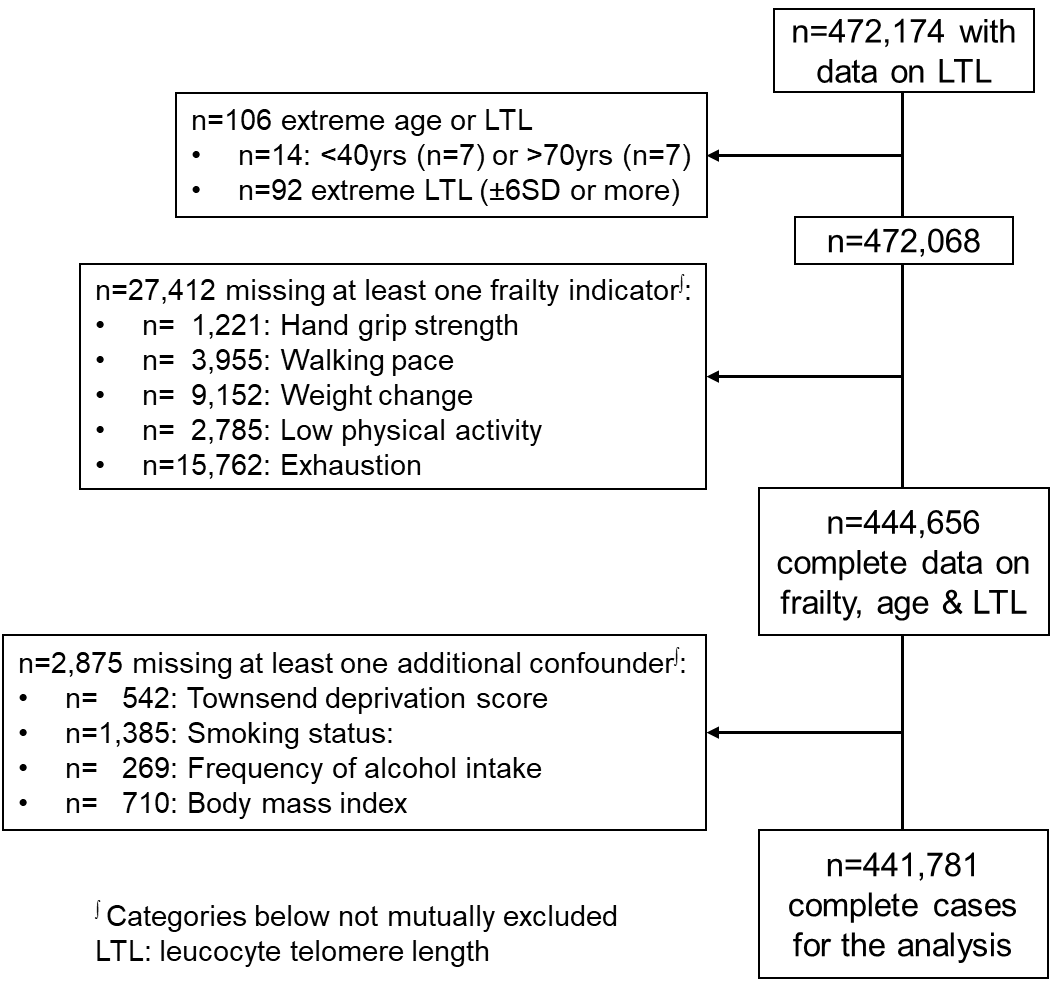


**Supplementary Figure 2: Participants’ distribution by frailty status over age, as occurred from the observed data and the fitted values from model M5 (~age+age^2^+LTL+LTL^2^), for average LTL (0SD).**


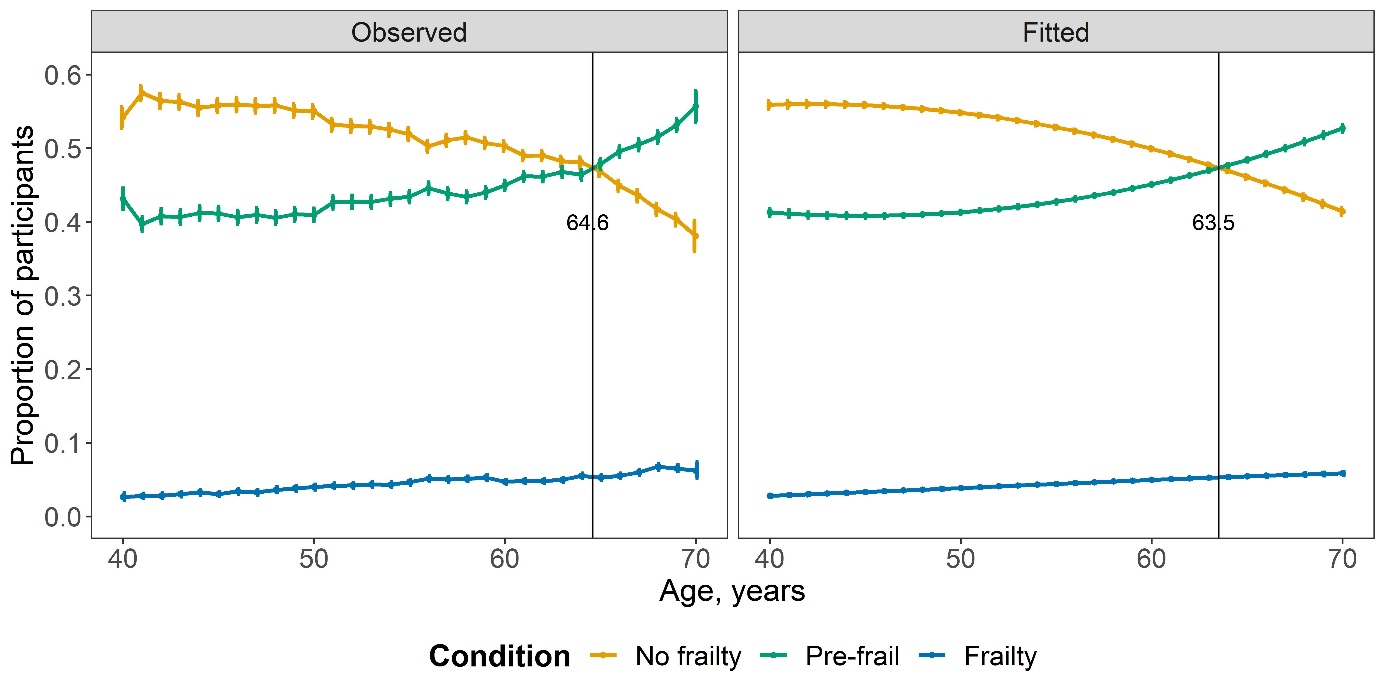

Supplement: Supplementary file 1 — Table S1: Adjusted relative risk ratios (RRR) and 95% confidence intervals (95% CI) from a multinomial regression model∫ of age, sex and leucocyte telomere length on number of frailty indicators. Table S2: Relative risk ratios (RRR) and 95% confidence intervals (95% CI) from multinomial logit models of age and leucocyte telomere length on frailty. Table S3: Adjusted odds ratios (OR) and 95% confidence intervals (95% CI) from a generalized ordinal model of age and leucocyte telomere length on frailty. Table S4: Adjusted relative risk ratios (RRR) and 95% confidence intervals (95% CI) from multinomial logit models of age, leucocyte telomere length (LTL) and sex on frailty. Table S5: Adjusted relative risk ratios (RRR) and 95% confidence intervals (95% CI) from a multinomial logit model on frailty, excluding n = 2,612 UKB participants with telomere length beyond ±3SDs (n = 439,420 with complete data). Figure S1: Flow of participants in the study. Figure S2: Participants' distribution by frailty status over age, as occurred from the observed data and the fitted values from model M5 (~age+age2 + LTL + LTL2), for average LTL (0SD). [file JCSM-13-1741-s001.docx]
